# Supplementary figures and images for: Comprehensive Analysis of Age- and Sex-Related Expression of the Chaperone Protein Sigma-1R in the Mouse Brain
Source: Brain Sci. 2024 Aug 30;14(9):881. doi: 10.3390/brainsci14090881 (PMC11430507; doi:10.3390/brainsci14090881)

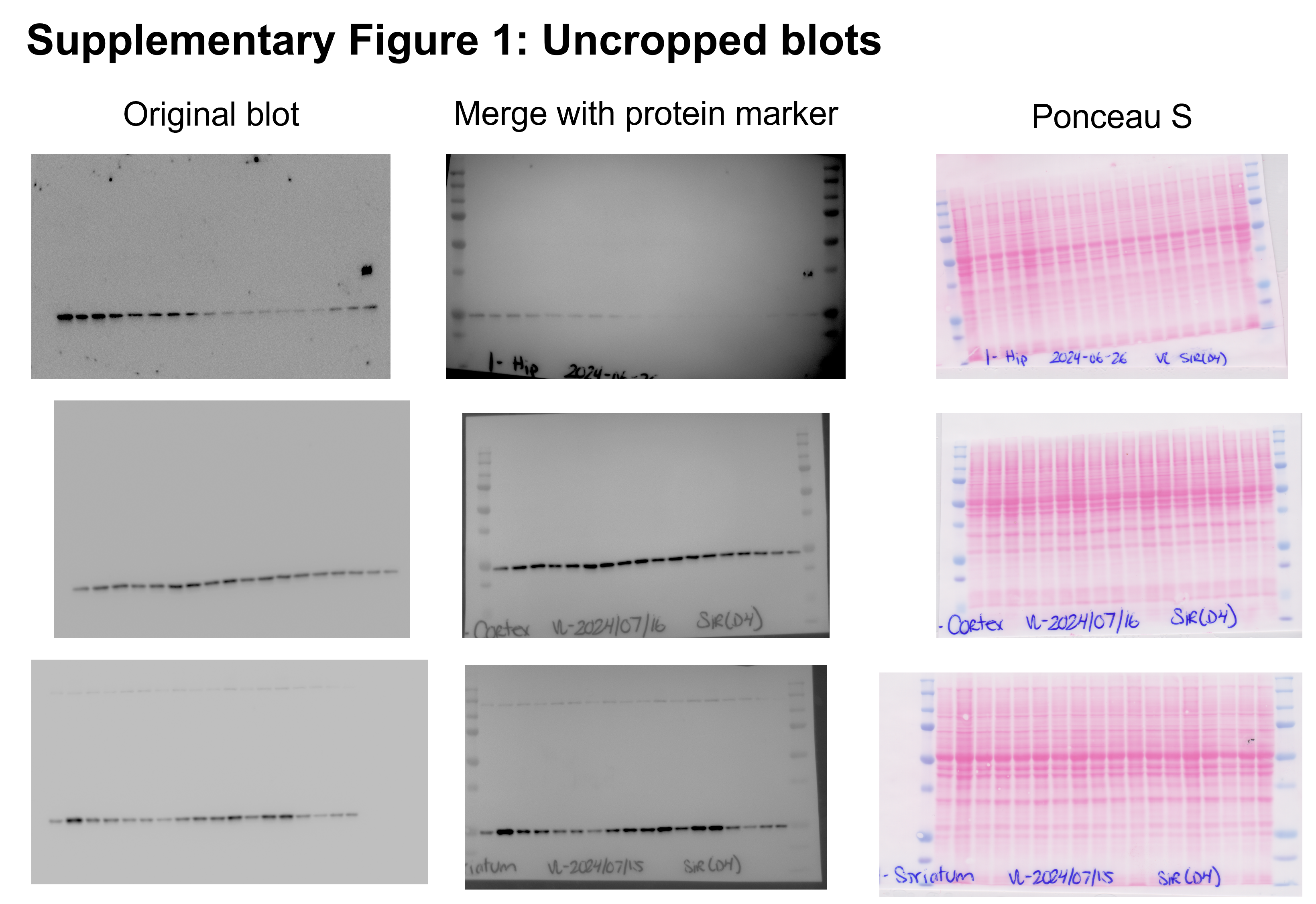

Supplement: Supplementary file 1 [file brainsci-14-00881-s001.zip › FigureS1.tif]

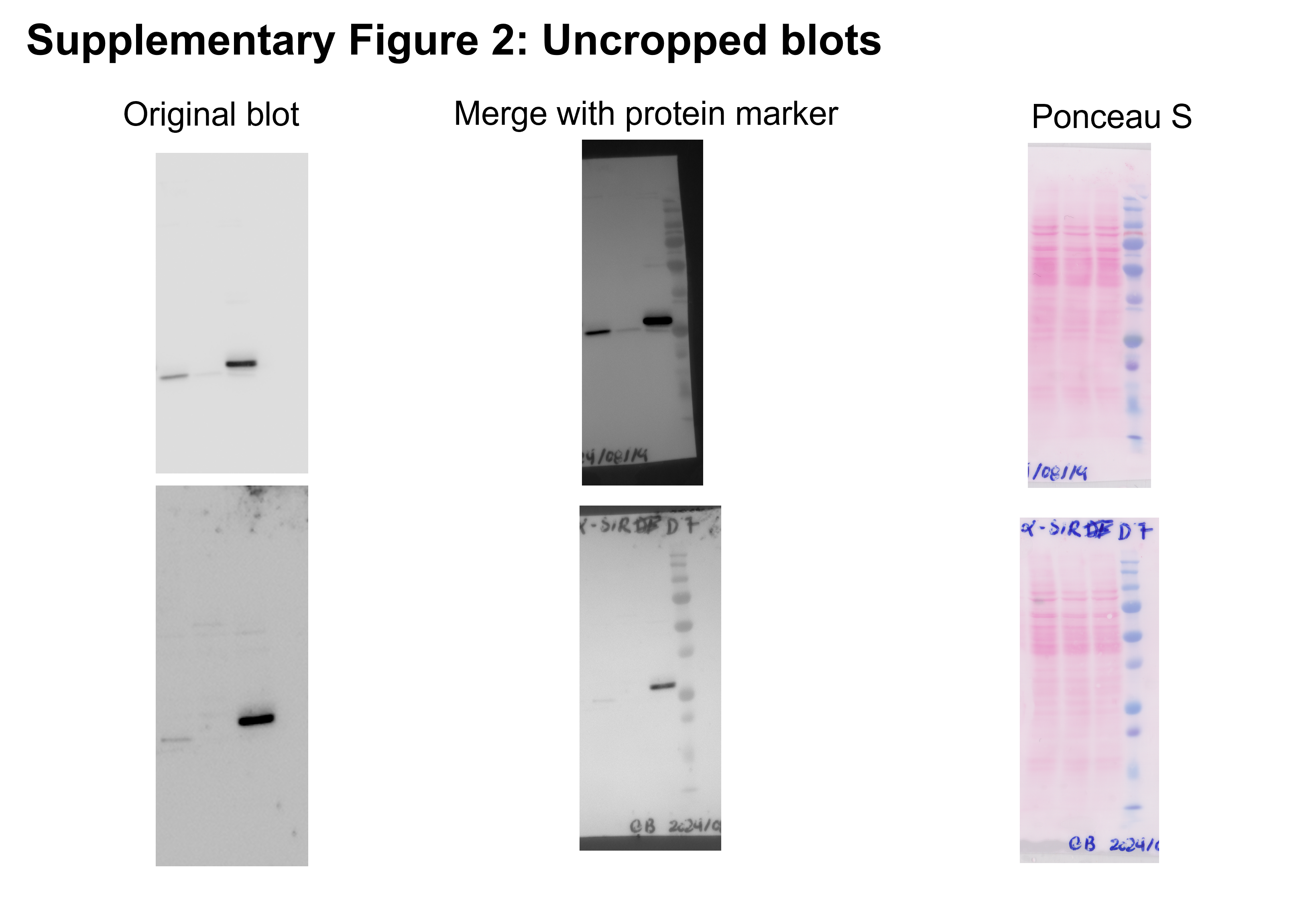

Supplement: Supplementary file 1 [file brainsci-14-00881-s001.zip › FigureS2.tif]
